# Supplementary material for: SciDaSynth: Interactive Structured Data Extraction From Scientific Literature With Large Language Model
Source: Campbell Syst Rev. 2025 Nov 3;21(4):e70073. doi: 10.1002/cl2.70073 (PMC12581027; doi:10.1002/cl2.70073)
Supplement: Supplementary file 1 — Supplementary Information [file CL2-21-e70073-s001.pdf]

PDF-XChange Editor Click to BUY NOW! www.pdf-xchange.com
PDF-XChange Editor Click to BUY NOW! www.pdf-xchange.com

A PROMPTS

We have leveraged LLMs to perform a variety of tasks on the content of a PDF document, including extraction, structuring, description, and question-answering. We present the prompts as follows.

A.1 Data Structuring

Data Structure Designs

Given the following question, design a structured data format to represent the answer:
Question: {*question*}.
Your task:
1. Carefully analyze the question to identify ONLY the specific information explicitly requested.
2. Design a table structure with columns that directly correspond to the requested information.
3. Provide the structure in a "record" format: [{{"*column_1*" : "*value_description*","*column_2*" : "*value_description*"}}]
4. Ensure all dictionary objects in the list share the same set of columns. 5. Use clear and descriptive names for the columns.
6. Avoid nested structures or hierarchical data - keep everything flat.

Guidelines:
- Choose column names that are self-explanatory and follow a consistent naming convention.
- Create columns ONLY for information directly mentioned or clearly implied in the question.
- Do NOT add columns for information that might be related but is not specifically asked for.
- Describe the expected values for each column (e.g., data type, format, range, units). - For columns with multiple possible values:
Create boolean columns for each option (e.g., "has_feature_X", "has_feature_Y"). * Or use a numeric scale to indicate presence/absence or degree (e.g., 0-5 scale). - For columns with a limited set of possible values, list all possible options.
- For numerical values (e.g., size, length, weight), specify the unit of measurement if relevant.
- For date/time values, specify the expected format.
Formulate your response as a JSON object containing the designed structure.

Ensure your structure capture all relevant information from the question, while also being flexible enough to accommodate various possible answers.

A.2 Document Processing

A.2.1 Meta Information Extraction

This prompt is used to extract the meta information in a PDF document, such as its title, abstract, year, and authors.

Meta Information Extraction

You should extract the meta information of the given paper. This is the paper content: {*paper*}.

Besides, the information you need to extract includes the following keys: "Title", "Abstract", "Year", "Author", "Journal/Conference", "ISSN", "Volume", "Issue", "Page", "DOI", "Link", "Publisher", "Language". For the page, please use the format like "12-15", "134-145". If there is only one page, the format can be "145", "1345". When there is no such information about a key, you just return the "none" as the value of the key, but you should make sure there is no such information. You should try your best to retrieve the information and reduce the occurrence of "none".

{*format_instructions*}

A.2.2 Table Identification

This prompt is leveraged to identify and extract tables embedded within a PDF document. However, the output of this prompt is a string representation of the table data, rather than a structured tabular format such as a CSV or JSON.

PDF-XChange Editor
Click to BUY NOW!
www.pdf-xchange.com

[illegible]ble Extraction

I will give you a page of a pdf file. You need first to judge whether there is any table in the page content. Then you need to extract the original information of the table from the page content. The following is the page content: {*page_content*}

If yes, just tell me the answer through the JSON format which includes the following keys: *table_name* and *table_content*.

Store all the JSON in a list through "[ ]". Besides, *table_name* is the Table order, such as Table 1, Table 2, and Table 3.

Note that you should tell me the related region of this table (raw data) from the page content without any processing in the *table_content*. Besides, you shouldn't output any other things (such as 'yes' or many explanations). That means, you just need to tell me the final output in JSON format in your response.
If not, just tell me "no".

A.2.3 Table Extraction

This prompt is leveraged to organize the tables extracted from a PDF document. Specifically, it takes the string representation of tables extracted in the previous extraction step and transforms it into a CSV format.

Table Structuring

I will give you a table content. You need to organize it in a CSV format. This is the step:
(1) You should determine the column names.
(2) You should fill in all the data in the corresponding column and row.

There are some points you should pay attention to:
(1) Don't leave out any of the information I gave you, you should organize all my information into a table for me.
(2) Be careful to "\n". If \n exists, there are two kinds of scenarios. First of all, it may be too long resulting in a branch, this time the front and back are actually one and the same. If you find that \n before and after can not form a whole, that is a nested table. the front column name is the parent column name of the back column name. At this time, you should add a parent column name. You should pay special attention when composing column names. You can use line breaks to notice which names are in a column. Here are a few different examples:
(a) ***example1****: For the column name message "Tempo de estocagem (dias)\n 0 55 90 145 180 235 280 360", you should pay special attention to the fact that there is an \n after Tempo de estocagem (dias), so this could mean that The column names 0 55 90 145 180 235 280 360 are sub-columns of Tempo de estocagem (dias). At this point you need to organize into: Tempo de estocagem (dias), Tempo de estocagem (dias), Tempo de estocagem (dias), Tempo de estocagem (dias), Tempo de estocagem (dias), Tempo de estocagem (dias), Tempo de estocagem (dias), Tempo de estocagem (dias), Tempo de estocagem (dias), Tempo de estocagem (dias) 0, 55, 90, 145, 180, 235, 280, 360. There are the column names at the previous level and column names at the next level, respectively.* ***There are more examples of this****: input: All-trans-b-caroteneb(mg/g DM) 13-cis-b-carotene Retention of\nall-trans -b-carotene (%)d\n(mg/g DM)c(% of total b-carotene): thoughts: Retention of \nall-trans -b-carotene (%) can be thought of as turning a row instead of two columns. \n(mg/g DM)c (% of total b-carotene) is a sub-column, and since it can be seen that 13-cis-b-carotene has no units, (mg/g DM)c and (% of total b-carotene) should be sub-columns of 13-cis-b-carotene. So the final column name should be organized as: output: All-trans-b-caroteneb (mg/g DM), 13-cis-b-carotene (mg/g DM)c, 13-cis-b-carotene (% of total b-carotene), Retention of all-trans-b-carotene (%)d*
(b) ***example2****: Sometimes the row breaks don't necessarily represent a relationship between the column name and the subcolumn name, such as the following: TPO2 a 23 °C, 1 atm(1) \n (mL (CNTP).m-2.dia-1). It may just be that the data is too long to be a unit. This time TPO2 a 23 °C, 1 atm(1) (mL (CNTP).m-2.dia-1) is one unit.*
(3) Note some of the special symbols such as ±.
(4) You need to ignore some special symbols, such as Unicode code point representations(e.g., /uni0394, /uni00A0).
(5)You should use "" to wrap every cell.
(6) Sometimes there will be redundant spaces, and you need to deal with those depending on the context. For example, there may be many spaces in "16 ± 0.6" due to noise, but they actually represent "16±0.6".

This is the content of my table: {*table_information*}
Tell me the answer in JSON format, including keys "table_caption" and "table_content", while "table_content" should be in string of CSV format.

A.3 Figure Description

This prompt is used to generate insights and descriptions for the figures that have been extracted from a PDF document.

Figure Description

"**role**": "user",
"**content**": [
{
"**type**": "text",
"**text**": f"I will give you a figure in the paper. Besides, I will also give you the caption of this figure. You should describe the data insight in this figure based on the caption. The more detailed the description, the better. This is the caption: {*caption*}."
},
{
"**type**": "image_url",
"**image_url**":
{"**url**": f"data:image/jpeg;base64,{*base64_image*}"}
}
]

B User Study Ratings

User study questionnaire ratings are presented in Figure 6.

C Item-wise Data Extraction Performance

We also computed the performance of different systems for Datasets I and II for each dimension to be extracted. The dataset I results are reported in Table 2. The dataset II results are reported in Table 3. The inter-rater agreement between two raters for individual data dimensions in the two datasets by method is summarized in Table 4.

Table 2: Dataset I: Per-item average performance distribution across different systems.

Category	Method	Correct (%)	Partial (%)	Incorrect (%)
Crop type	SciDaSynth	95.8	2.5	1.7
	Baseline A	93.8	4.2	2.1
	Baseline B	83.3	8.3	8.3
Micronutrient	SciDaSynth	93.8	4.2	2.1
	Baseline A	87.5	8.3	4.2
	Baseline B	85.4	4.2	10.4
Raw value	SciDaSynth	83.3	8.3	8.3
	Baseline A	83.3	8.3	8.3
	Baseline B	72.8	15.0	12.2
Unit	SciDaSynth	85.4	8.3	6.3
	Baseline A	85.4	7.9	6.7
	Baseline B	75.0	12.5	12.5

Table 3: Dataset II: Per-item average performance distribution across different systems.

Category	Method	Correct (%)	Partial (%)	Incorrect (%)
Model name	SciDaSynth	98.8	0.6	0.6
	Baseline A	95.0	3.2	1.8
	Baseline B	90.6	6.3	3.1
Model size	SciDaSynth	94.4	2.5	3.1
	Baseline A	93.0	3.9	3.1
	Baseline B	87.5	5.6	6.9
Pretrained data scale	SciDaSynth	90.6	4.4	5.0
	Baseline A	89.4	5.6	5.0
	Baseline B	81.3	8.8	10.0
Hardware specs	SciDaSynth	90.0	8.1	1.9
	Baseline A	87.5	5.0	7.5
	Baseline B	84.4	5.0	10.6

PDF-XChange Editor
Click to BUY NOW!
www.pdf-xchange.com
PDF-XChange Editor
Click to BUY NOW!
www.pdf-xchange.com

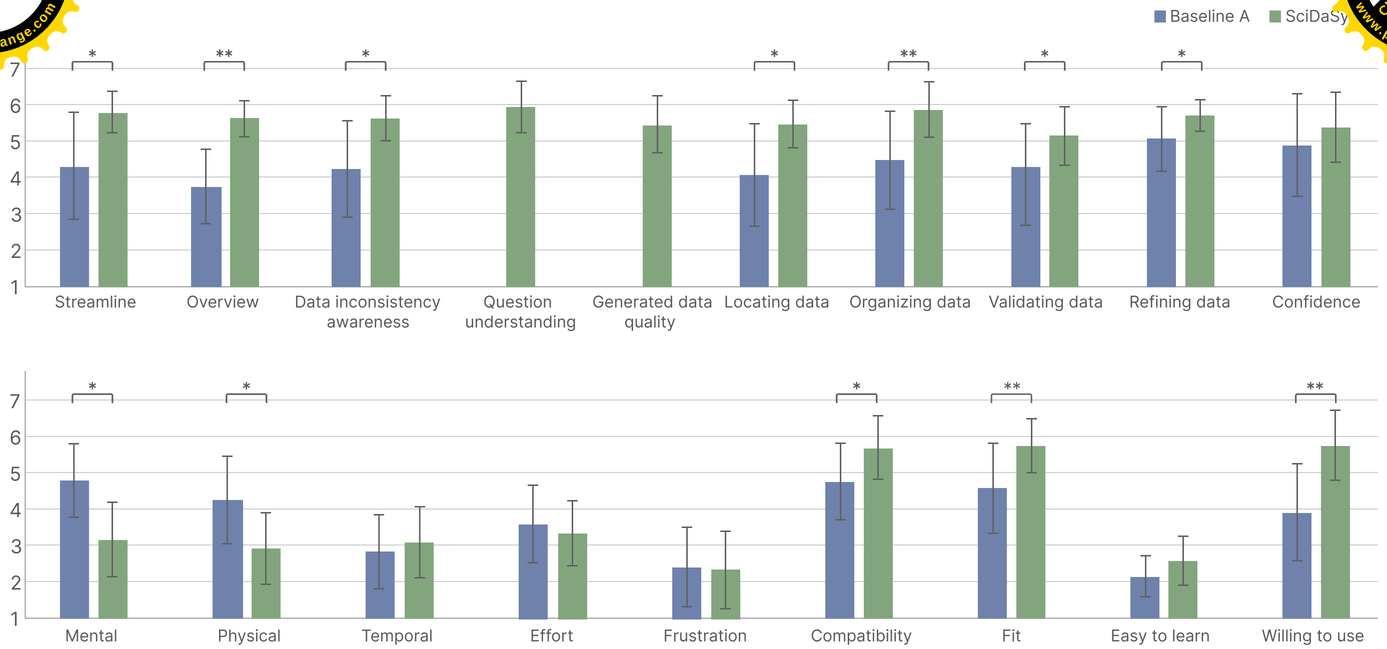

Fig. 6: User study questionnaire results for both Baseline A and *SciDaSynth*. The first row of items compared the ratings regarding the effectiveness in streamlining data extraction workflow, gaining an overall understanding of the paper collection, awareness of data inconsistencies, question understanding, perceived generated data quality, data locating, organization, validation, refinement, and confidence in the final data table. The second row compared the questionnaire items adapted from the NASA Task Load Index and the technology acceptance model. All ratings were on a 7-point scale. For ratings: "Mental", "Physical", "Temporal", "Frustration", "Easy to learn", the lower the ratings, the better. For all other ratings, the higher the ratings, the better. **: $p < 0.01$, *: $p < 0.05$.

Table 4: Inter-rater Cohen's κ by dataset and method

Dataset	Method	κ
I (Nutrition)	SciDaSynth	0.763
	Baseline A	0.721
	Baseline B	0.775
II (LLM)	SciDaSynth	0.738
	Baseline A	0.767
	Baseline B	0.842
